# The effects of surgeon handedness on the efficacy and safety of proximal femoral nail antirotation fixation for intertrochanteric femur fractures in elderly patients: A single Center’s experience

**DOI:** 10.3389/fbioe.2025.1548823

**Published:** 2025-03-19

**Authors:** Xiao-Feng Liu, Yong-Qiang Zheng, Liang Lin, Zhen-Yu Lin, Hong-Peng Zhang, Xiao-Peng Huang, Ze-Feng Wang, Yang-Zhen Fang, Wen-Ming Zhang, Xin-Yu Fang, Jin-Shan Zhang

**Affiliations:** ^1^ Department of Orthopaedic Surgery, The First Affiliated Hospital of Fujian Medical University, Fuzhou, China; ^2^ The First Clinical College of Fujian Medical University, Fuzhou, China; ^3^ Department of Orthopedics, Jinjiang Municipal Hospital (Shanghai Sixth People’s Hospital Fujian), Quanzhou, Fujian, China; ^4^ Clinical Research Center for Orthopaedic Trauma and Reconstruction of Fujian Province, Quanzhou, Fujian, China; ^5^ Department of Orthopaedic Surgery, National Regional Medical Center, Binhai Campus of the First Affiliated Hospital, Fujian Medical University, Fuzhou, China; ^6^ Fujian Provincial Institute of Orthopedics, The First Affiliated Hospital, Fujian Medical University, Fuzhou, China

**Keywords:** handedness, intertrochanteric femur fracture, PFNA, safety, efficacy

## Abstract

**Background:**

Handedness refers to the lateral preference of using one hand more than the other. Surgeon handedness has been widely discussed in operative surgery and could cause clinical discrepancy. However, in the use of proximal femoral nail antirotation (PFNA) for the treatment of intertrochanteric femur fracture (IFF), the effect of handedness on clinical outcomes is easily overlooked. In fact, when right-handed surgeons operate on right-sided IFF patients, due to the specific nature of the surgical site, they have to use their left hand to perform the opening at the proximal end of the femur and insert the main nail. This study aimed to investigate the influence of surgeon handedness on the efficacy and safety of PFNA fixation for elderly patients with IFF.

**Methods:**

A retrospective analysis was conducted on the basic data of 182 elderly patients with IFF who underwent surgical treatment in our department from January 2020 to December 2022 and had a minimum follow-up duration of 1 year. Equal numbers of left and right PFNA fixation were performed by four surgeons, two right-handed and two left-handed. Dominant group refers to a left IFF for a right-handed surgeon and a right IFF for a left-handed surgeon. Otherwise, it is called a non-dominant group. The patients were divided into the dominant group (90 cases) and the non-dominant group (92 cases), and differences between the two groups were analyzed.

**Results:**

In terms of surgical safety, the dominant group had significantly shorter average operation time and lower blood loss compared to the non-dominant group (P < 0.05). In the final follow-up, the average Harris score of the dominant group was 84.60 ± 4.35, and that of the non-dominant group was 82.63 ± 4.98. The Harris score of the dominant group was significantly higher than that of the non-dominant group (P < 0.05). According to the 1-year follow-up results, there were 86 survivors and 4 deaths in the dominant group, and 80 survivors and 12 deaths in the non-dominant group. Although the non-dominant group (13.04%) had a higher mortality rate than the dominant group (4.44%), the difference between the two groups was not statistically significant (P > 0.05).

**Conclusion:**

Surgeon handedness is a factor that influences the efficacy and safety of PFNA fixation for patients with IFF.

## Introduction

IFF refers to fractures occurring between the base of the femoral neck and the greater trochanter, mainly affecting elderly individuals with osteoporosis and characterized by a high incidence, mortality rate, and disability rate (Li et al., 2014). At present, the influential factors affecting the efficacy and safety of IFF surgery have been reported in the literature, such as age, sex, American Society of Anesthesiologists (ASA) grade, number and type of combined medical diseases, post-injury complications, operation time, anti-osteoporosis treatment, and pre-fracture walking ability (Iosifidis et al., 2016; Wang et al., 2022). Surgery is the most effective treatment for elderly patients with IFF, and PFNA fixation is a commonly used technique.

Previous studies have reported significant effects of the surgeon’s handedness on surgical outcomes. Handedness refers to the lateral preference of using one hand more than the other (Hanna et al., 1997). According to the literature, the handedness of surgeons is widely discussed in laparoscopic abdominal surgery (Tchantchaleishvili and Myers, 2016), but it is seldom mentioned in orthopedic surgeries. Given the symmetrical distribution of the human skeleton, the impact of a surgeon’s handedness on orthopedic surgery may be greater than on non-orthopedic procedures. When a right-handed surgeon operates on a patient with a right IFF, they have to use their less skilled left hand to perform procedures such as opening with a pointed awl and inserting the main nail. Similarly, a left-handed surgeon operating on a left-sided IFF patient may encounter a similar situation. Currently, it is unclear whether the handedness of the surgeon affects the effectiveness and safety of PFNA fixation surgery in patients with IFF. In this paper, ‘dominant side’ refers to a left IFF for a right-handed surgeon and a right IFF for a left-handed surgeon.

The aim of this study was to investigate whether the handedness of surgeons affects the efficacy and safety of PFNA fixation surgery in patients with IFF. A retrospective analysis of clinical data from 182 IFF patients who underwent treatment in our hospital from January 2020 to December 2022 was conducted to consider this potential significant factor.

## Methods

### Study population

**FIGURE 1:**
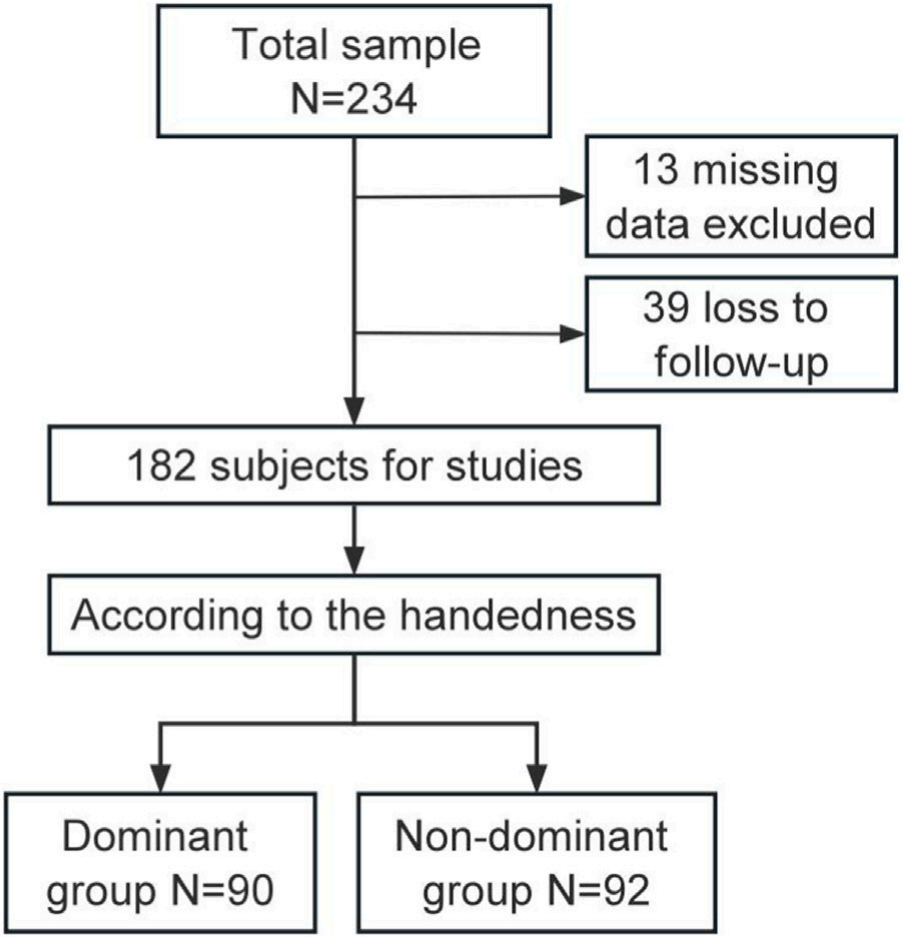
Flow chart of the study cohort.

This study was approved by the Medical Ethics Committee of our hospital (jjsyyyxll-2022110). This study was conducted in accordance with the principles of the Declaration of Helsinki. Informed consent was obtained from all patients. We conducted a retrospective analysis of patients with IFF who were admitted to our institution and had a follow-up period of more than 1 year from January 2020 to December 2022. A total of 234 patients suffered from IFF were enrolled. Of these, 52 patients were excluded, resulting in a final sample of 182 patients. Figure 1 presents a flow diagram of the study cohort. Four orthopedic surgeons participated in the study, with two being right-handed and the other two being left-handed. The basic information and stated handedness for each surgeon were:

Surgeon 1: 41 years old, Associate Chief Physician, RHD, performs over 200 cases per year.
Surgeon 2: 52 years old, Chief physician, RHD, performs over 300 cases per year.
Surgeon 3: 40 years old, Associate Chief physician, LHD, performs over 200 cases per year.
Surgeon 4: 49 years old, Chief physician, LHD, performs over 300 cases per year.

### Inclusion criteria

Inclusion criteria were as follows: (1) age ≥60 years, (2) all patients in the sample had mild injuries, such as fractures resulting from falls with confirmed diagnoses based on X-ray or CT reports, (3) diagnosed osteoporosis, (4)underwent surgical treatment, (5) complete medical records and follow-up data. Exclusion criteria were as follows: (1) multiple fractures, (2) high-energy injuries, such as motor vehicle accidents, falls from heights, or impact injuries, (3) hip pathologic fractures caused by primary or metastatic tumors.

### Data collection

Clinical data of patients, including age, gender, admission date, discharge date, fracture location, fracture classification, duration of surgery, blood loss, need for blood transfusion, reduction status, and length of hospital stay, were obtained through the electronic medical record system. Patient survival status was assessed through outpatient visits or telephone follow-up, and the mortality rate at 1 year after fracture was calculated. The Harris score at the last follow-up visit was recorded.

A total of 182 patients were included in this study, including 58 males and 124 females, with ages ranging from 60 to 100 years (mean age: 82.45 ± 9.19 years). The Edinburgh Handedness Inventory was used to assess hand dominance for the surgeons in this study (Oldfield, 1971). According to the difference in surgical side and surgeon’s dominant hand, all patients were divided into the dominant group and the non-dominant group. The dominant group refers to cases where the surgical side is opposite to the surgeon’s dominant hand, while the non-dominant group refers to cases where the surgical side is the same as the surgeon’s dominant hand. The dominant group consisted of 90 patients, including 28 males and 62 females. The non-dominant group consisted of 92 patients, including 30 males and 62 females.

### Surgical technique

We followed a standard surgical procedure. The PFNA surgical instruments used in this study were provided by Zhengtian. The patient was placed in a supine position on a traction bed in the orthopedic department. A soft cushion was placed under the affected side’s buttocks to elevate it, while the unaffected side was flexed and abducted at the hip. Under fluoroscopic guidance using a C-arm X-ray machine, closed reduction was performed through traction, abduction, external rotation, adduction, and internal rotation. After satisfactory reduction, a vertical incision of 3–5 cm was made extending proximally from the tip of the greater trochanter. The muscles were bluntly dissected until the tip of the greater trochanter was reached. With fluoroscopic assistance, a guide pin was inserted from the lateral side, directed inward and forward. Once satisfactory position was confirmed on fluoroscopy, the medullary canal was reamed. The PFNA (proximal femoral nail antirotation) main nail was inserted, and the depth and anteversion angle were adjusted. The proximal helical blade was inserted along the direction of the femoral neck, impacted, and confirmed to be in the correct position. The distal locking screws were then inserted and secured with the end cap. Positioning was confirmed again under fluoroscopy. The incision was sutured layer-by-layer and surgery was then concluded.

### Perioperative management

After admission, relevant preoperative routine examinations were completed. If there were any comorbidities, appropriate treatment for the underlying condition was provided. Routine prophylactic antibiotics were administered 30 min before surgery. Postoperatively, prophylactic antibiotics were continued for 24 h, and anticoagulant medications were used to prevent lower limb thrombosis. For patients with more severe conditions, transfer to the intensive care unit (ICU) or orthopedic ICU was considered if necessary. Close monitoring of vital signs, especially cardiovascular and pulmonary function, was carried out, and postoperative care was intensified.

### Postoperative rehabilitation

The rehabilitation protocol was standardized. On postoperative day 1, ankle pumps and quadriceps, hamstring, and other muscle length exercises were performed. Bilateral hip abduction and adduction exercises were also conducted, along with passive or active range of motion exercises for the hip and knee joints. On postoperative day 2, straight leg raising exercises were added. On postoperative day 3, lateral straight leg raising exercises were introduced. Weight-bearing was adjusted according to the internal fixation status. All patients were followed up at 6 weeks, 3 months, and 1 year after surgery. During each follow-up, the surgical team assessed joint function scores and obtained pelvic and anteroposterior and lateral X-rays of the femur.

## Clinical outcomes

### Evans classification criteria

IFF were classified into subtrochanteric and pertrochanteric fractures according to the Evans classification (Evans, 1949). (1)Type one subtrochanteric fractures were further divided into four subgroups: 1.1 - non-displaced fractures (stable fractures); 1.2 - partially displaced fractures with intact greater and lesser trochanters; 1.3 - lesser trochanter avulsion or isolated greater trochanter as a separate fragment with displacement and varus deformity; 1.4 - fractures involving both the subtrochanteric region and the greater and lesser trochanters with varus deformity. (2)Type two pertrochanteric fractures had a fracture line extending from below the greater trochanter obliquely inward and upward to above the lesser trochanter.

### Reduction status

Classification of reduction results based on postoperative pelvic X-ray (Chang et al., 2015): (1)Positive Medial Cortex Support (PMCS): The proximal femoral head-neck fragment is shifted inward to the inner edge of the distal femoral shaft fragment, with the inner cortex of the head-neck fragment positioned slightly above (by one cortical thickness) the inner cortex of the femoral shaft. (2)Neutral Position (NP): The inner cortex of the head-neck fragment anatomically contacts the femoral shaft fragment. (3)Negative Medial Cortex Support (NMCS): The head-neck fragment is shifted outward to the inner edge of the femoral shaft fragment, resulting in loss of medial cortex support in the femoral shaft.

### Statistical analysis

Data were analysed using IBM SPSS26.0. Continuous data were presented as mean ± standard deviation. When the data followed a normal distribution, independent samples t-test was used to compare the two groups. For data that did not follow a normal distribution, the Mann-Whitney U test was used. Categorical data were analyzed using the chi-square test. If all expected counts were greater than or equal to five and the total sample size was greater than or equal to 40, the Pearson chi-square test was used. If any expected count was less than five but greater than or equal to 1, and the total sample size was greater than or equal to 40, the chi-square test with continuity correction was used. A two-sided P < 0.05 was considered statistically significant.

## Results

### Comparison of preoperative indicators between the dominant group and non-dominant group

**FIGURE 2:**
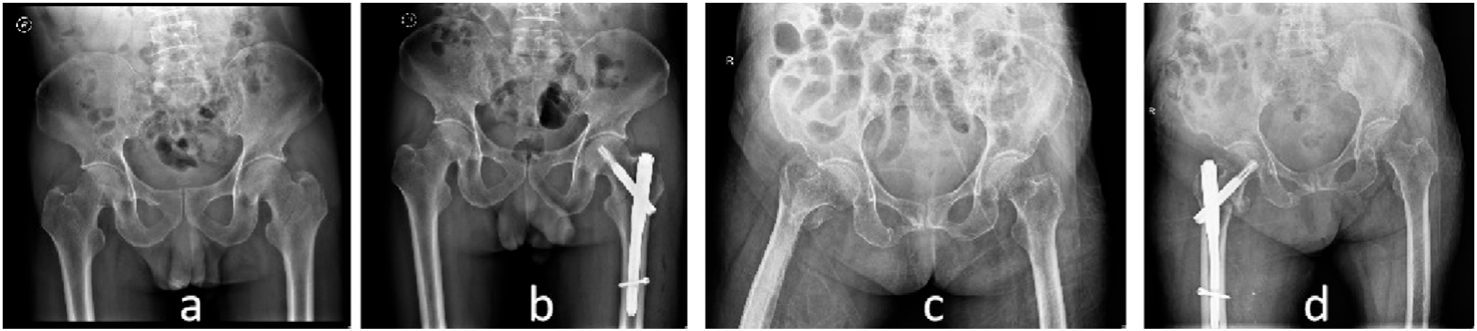
X-ray images of two patients. Case 1 is an 80-year-old female with a stable fracture. **(A)** preoperative pelvis radiograph and **(B)** postoperative pelvis radiograph. Case 2: A 102-year-old female with unstable fracture. **(C)** preoperative pelvis radiograph and **(D)** postoperative pelvis radiograph.

The dominant group consisted of 90 cases with a mean age of 82.73 ± 8.24 years, while the non-dominant group consisted of 92 cases with a mean age of 82.17 ± 10.07 years. There was no statistically significant difference in age between the two groups (P > 0.05). In the dominant group, there were 28 males and 62 females, while in the non-dominant group, there were 30 males and 62 females. There was no statistically significant difference in gender distribution between the two groups (P > 0.05). According to the Evans classification, the dominant group had 2 cases of type 1.1, 30 cases of type 1.2, 26 cases of type 1.3, and 32 cases of type 1.4. Stable fractures included type 1.1 and 1.2, totaling 32 cases, while unstable fractures included type 1.3 and 1.4, totaling 58 cases. In the non-dominant group, there were 10 cases of type 1.1, 18 cases of type 1.2, 28 cases of type 1.3, and 36 cases of type 1.4. Stable fractures included type 1.1 and 1.2, totaling 28 cases, while unstable fractures included type 1.3 and 1.4, totaling 64 cases. There was no statistically significant difference in fracture types between the two groups (P > 0.05). Figure 2 shows two typical examples of stable and unstable fractures from the study (Table 1).

**TABLE 1:** Comparison of preoperative indicators between the dominant group and non-dominant group.

	Dominant group (n = 90)	Non-dominant group (n = 92)	t/χ2 value	p value
Age	82.73 ± 8.24	82.17 ± 10.07	0.410	0.682
Sex			0.047	0.828
Male	28	30		
Female	62	62		
Evans classification			0.540	0.463
Stable	32	28		
Unstable	58	64		
Type 1.1	2	10		
Type 1.2	30	18		
Type 1.3	26	28		
Type 1.4	32	36		

### Comparison of intraoperative indicators between the dominant group and non-dominant group

**FIGURE 3:**
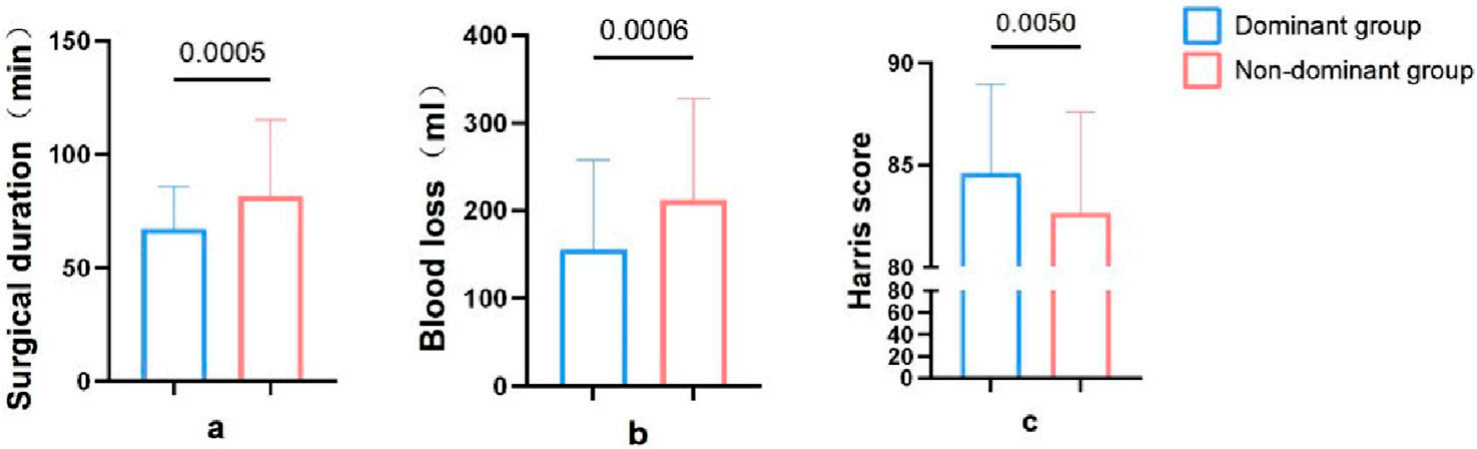
Comparison of intraoperative indexes between dominant group and non-dominant group. **(a)**, Surgical duration. **(b)**, Blood loss. **(c)**, Harris scores.

The dominant group had an average surgery time of 67.11 ± 18.66 min, while the non-dominant group had an average surgery time of 81.50 ± 33.79 min. The surgery time in the dominant group was significantly lower than the non-dominant group, with a statistically significant difference (P < 0.05) (Figure 3A).

The dominant group had an average blood loss of 156.22 ± 101.92 mL, while the non-dominant group had an average blood loss of 212.39 ± 114.63 mL. The blood loss in the dominant group was significantly lower than the non-dominant group, with a statistically significant difference (P < 0.05) (Figure 3B).

### Comparison of postoperative indicators between the dominant group and non-dominant group

The dominant group had an average postoperative hospital stay of 13.44 ± 6.86 days, while the non-dominant group had an average postoperative hospital stay of 13.70 ± 6.12 days. There was no statistically significant difference in the duration of hospital stay between the two groups (P > 0.05) (Table 2).

**TABLE 2:** Comparison of postoperative indexes between dominant group and non-dominant group.

	Dominant group (n = 90)	Non-dominant group (n = 92)	t/χ2 value	p value
Length of hospital stay postoperatively(d)	13.44 ± 6.86	13.70 ± 6.12	0.261	0.794
1-year follow-up	90	92	3.192	0.074
Death	4	12		
Survival	86	80		
Hb(g/l)				
First admission Hb level	103.93 ± 24.69	106.91 ± 22.83	0.846	0.399
Hb level in the first postoperative assessment	89.16 ± 17.07	92.02 ± 16.91	1.104	0.271
t value	4.256	4.814		
p value	<0.001	<0.001		
Blood transfusion			0.025	0.876
Yes	42	44		
No	48	48		
Reduction status			0.150	0.698
PMCS + NP	18 + 70	15 + 73		
NMCS	2	4		

During the 12-month follow-up period, the dominant group had 86 cases of survival and 4 cases of death, while the non-dominant group had 80 cases of survival and 12 cases of death. There was no statistically significant difference in survival rates between the two groups (P > 0.05).

In terms of hemoglobin (Hb) levels, the dominant group had an average preoperative Hb level of 103.93 ± 24.69 g/L, which decreased to an average postoperative Hb level of 89.16 ± 17.07 g/L. The difference in Hb levels before and after surgery was statistically significant (P < 0.05). Similarly, the non-dominant group had an average preoperative Hb level of 106.91 ± 22.83 g/L, which decreased to an average postoperative Hb level of 92.02 ± 16.91 g/L. The difference in Hb levels before and after surgery was statistically significant (P < 0.05). There was no statistically significant difference in preoperative Hb levels between the dominant and non-dominant groups (P > 0.05). Likewise, there was no statistically significant difference in postoperative Hb levels between the two groups (P > 0.05).

In the final follow-up, the dominant group had an average Harris score of 84.60 ± 4.35, while the non-dominant group had an average Harris score of 82.63 ± 4.98. There was a statistically significant difference in the Harris scores between the two groups at the final follow-up (P < 0.05) (Figure 3C).

In the dominant group, 42 patients received blood transfusion during or after surgery, while 48 patients did not. In the non-dominant group, 44 patients received blood transfusion, while 48 patients did not. There was no statistically significant difference in the number of transfused cases between the two groups (P > 0.05).

**FIGURE 4:**
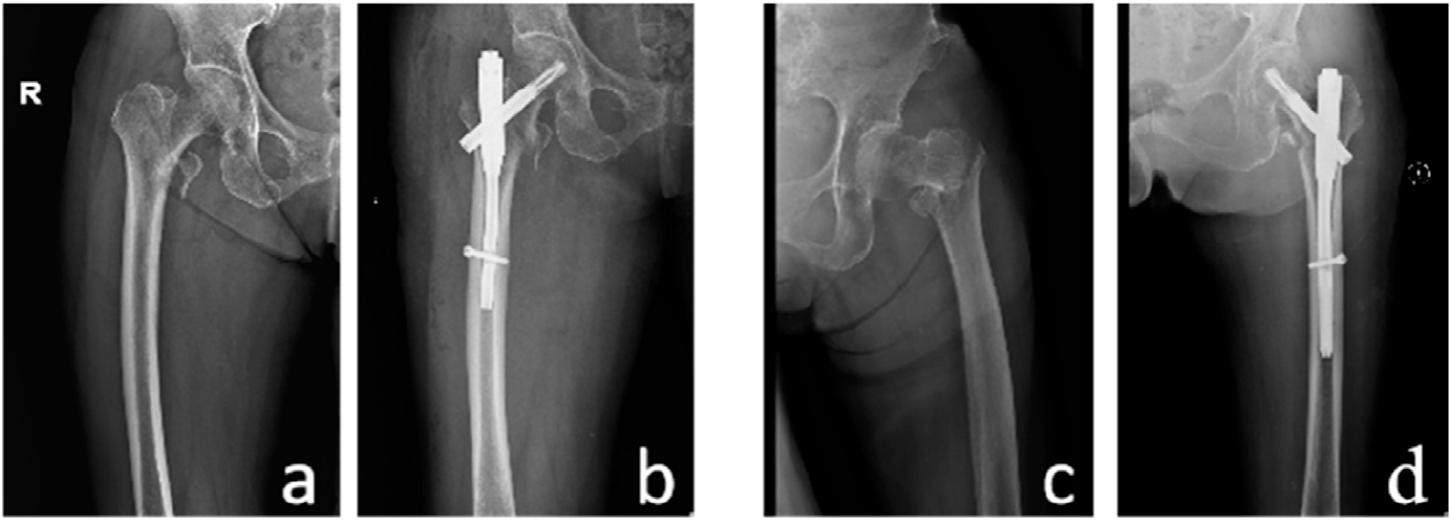
Two patients with unstable fractures. Case 1, an 80-year-old female. **(A)** Preoperative pelvic radiograph, **(B)** PMCS. Case 2, an 83-year-old female. **(C)** Preoperative pelvic radiograph, **(D)** NMCS.

In terms of achieving positive support in PFNA surgery, the dominant group had 88 cases with positive support and 2 cases with negative support, while the non-dominant group had 88 cases with positive support and 4 cases with negative support. The proportion of cases receiving positive support was higher in the dominant group than in the non-dominant group, but the difference was not statistically significant (P > 0.05). Figure 4 shows two typical examples of PMCS and NMCS from the study.

### Complications

**FIGURE 5:**
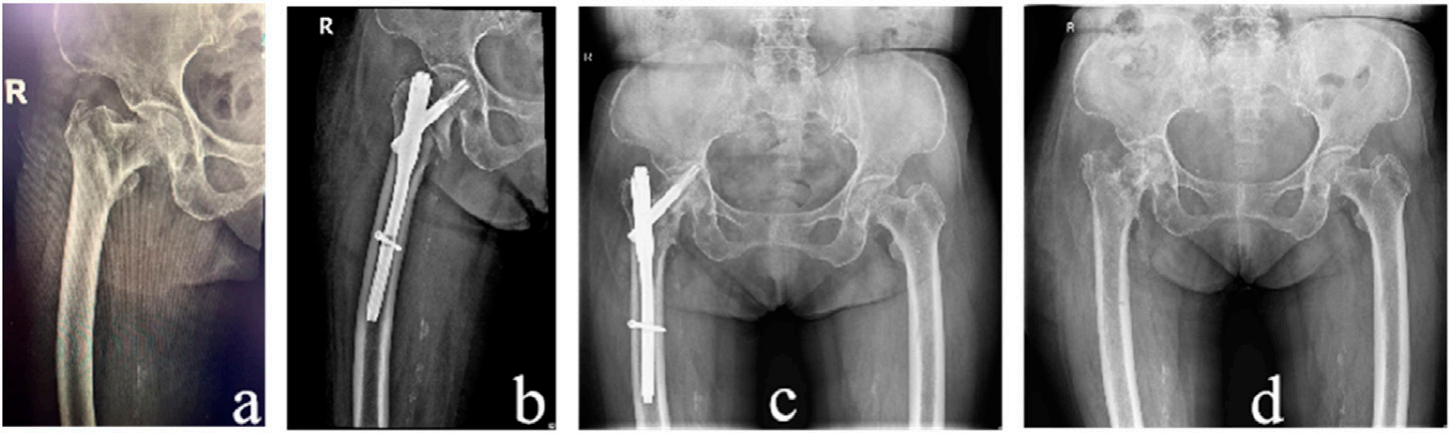
Female, 83-year-old, with postoperative malunion. **(A)** preoperative X-ray images, **(B)** postoperative X-ray indications of poor fracture reduction, **(C)** postoperative malunion, **(D)** removal of internal fixations.

A patient with an unstable fracture in the non-dominant group had an unstable vital sign due to poor intraoperative reduction, long operative time, and heavy bleeding, so the surgery was completed without a good reduction. Malunion occurred after the operation. However, this patient underwent active rehabilitation and functioned well after surgery, so revision surgery was not required (Figure 5).

**FIGURE 6:**
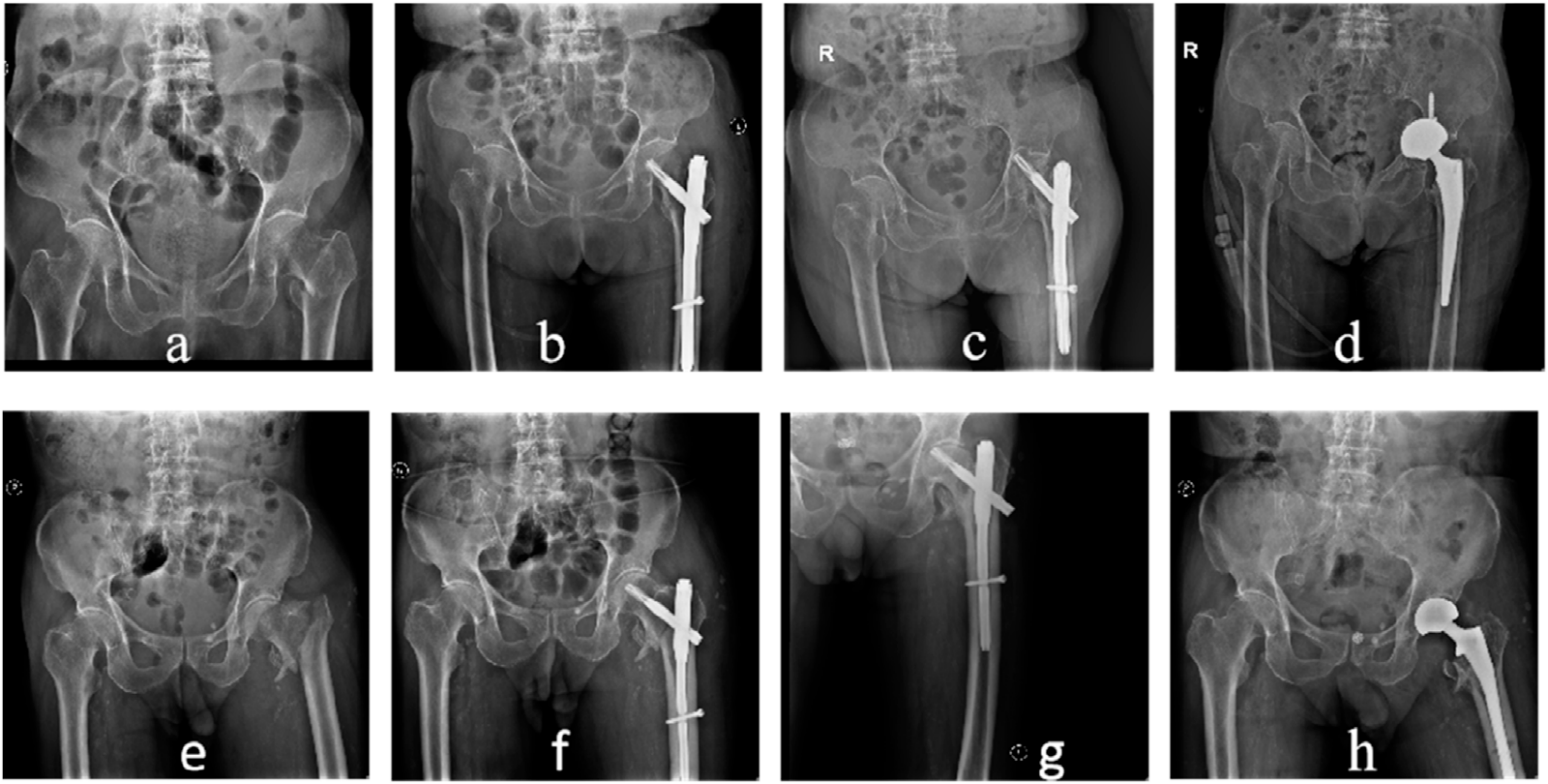
Two revision cases. Case 1, a 96-year-old female, was from the dominant group. **(A)** preoperative radiographs of the pelvis, **(B)** postoperative radiographs of the pelvis, **(C)** postoperative reexamination of the femoral head revealed that the screw had been punctured, and **(D)** revision surgery was performed. Case 2, an 89-year-old male from the non-dominant group. **(E)** preoperative radiograph of the pelvis, **(F)** postoperative radiograph of the pelvis, **(G)** postoperative reexamination revealed necrosis of the femoral head, **(H)** revision surgery was performed.

Femoral head necrosis and collapse of the femoral head occurred in two patients after surgery, resulting in continuous hip pain caused by screw nails penetrating the femoral head. The two patients were from the dominant group and the non-dominant group. Revision surgery was performed on these two patients, including total hip replacement in one patient and artificial femoral head replacement in the other. Both patients recovered well after surgery. Figure 6 shows these two typical cases in the research.

## Discussion

Our study concludes that the handedness of surgeons does impact the efficacy and safety of PFNA fixation surgery in patients with IFF. Although there was no significant difference in the 1-year mortality rate between the dominant group and the non-dominant group, patients in the dominant group had better fracture reduction, shorter surgical times, less intraoperative bleeding, and better functional scores at the 1-year follow-up compared to the non-dominant group.

PFNA fixation is one of the most commonly used surgical methods for treating IFF. However, due to the natural right-handed dominance of most people, right-handed surgeons may have to use their less skilled left hand during surgery to perform the proximal femur opening as well as inserting the main nail, since the left hand is obviously less skilled in controlling direction and force than the right hand. Therefore, performing surgery with the non-dominant hand may increase the surgical time and intraoperative blood loss, which could potentially impact patient outcomes and the safety of the procedure.

The shorter surgical times and reduced blood loss observed in the dominant group may have important long-term implications for patient care and clinical practice. Shorter surgical durations are generally associated with reduced exposure to anesthesia, lower risk of infection, and decreased physiological stress, all of which can contribute to faster recovery and improved postoperative mobility. Similarly, reduced intraoperative blood loss can minimize the need for transfusions, which has been linked to better outcomes, particularly in elderly patients with intertrochanteric fractures. These findings suggest that optimizing surgical conditions to mitigate the impact of handedness—such as through targeted ambidexterity training, the use of advanced imaging and navigation technologies, and preoperative planning—could lead to tangible benefits for patients. This is especially relevant for high-risk populations, such as elderly patients with osteoporosis, who are more vulnerable to complications and prolonged recovery. Future research with extended follow-up periods should explore whether these intraoperative advantages translate into improved long-term outcomes, including lower complication rates, better functional recovery, and higher patient satisfaction. Such insights would strengthen the evidence base for incorporating handedness considerations into clinical decision-making and surgical training programs.

The prevalence of right-handedness in the population ranges from 78% to 90% (Hardyck and Petrinovich, 1977; Peters et al., 2006; Sabharwal et al., 2020). Although there is currently no research on the influence of handedness on PFNA surgery for treating IFF fractures, in the past, some scholars have studied the correlation between handedness and the efficacy and safety of other orthopedic surgeries. Moloney et al. (1994) compared pre- and post-operative X-rays of 244 patients treated with sliding hip screws for hip fractures and found that all 12 cases of surgical failure occurred on the non-dominant side. Pennington et al. (2014) found that in hip arthroplasty, when surgeons operate on the non-dominant side, the anteversion angle of the acetabular component is smaller compared to the dominant side. Yaman and Acaroğlu (2014) and Akyoldaş et al. (2018) found that the rate of screw misplacement increased significantly when right-handed surgeons implanted upper thoracic pedicle screws on the left side of the patient compared to the right side. Some studies have investigated the impact of surgeon handedness on the outcomes of joint replacement surgery. In one study involving 94 patients undergoing unicompartmental knee arthroplasty, it was found that surgeon handedness might lead to malalignment of the femoral component (Cao et al., 2022). In a retrospective study involving 102 patients undergoing bilateral direct anterior approach total hip arthroplasty, it was found that surgeon handedness had a significant impact on the anteversion angle of the acetabular cup and the fill of the femoral component (Kong et al., 2020). Most studies have found some influence of handedness on surgical outcomes. However, there are also a few studies that have found no association between handedness and surgical efficacy. Berthold et al. (2020) found in a retrospective study involving 40 patients (44 shoulders) that the surgeon’s handedness did not affect the short-term efficacy of total shoulder arthroplasty.

In our study, although the surgeries were performed by experienced surgeons, we still found a impact of handedness on surgical outcomes. Handedness is a long-standing preference for using one hand over the other, and it is not easily changed overnight. In our study, there were no significant differences in gender, age, and fracture type distribution between the dominant and non-dominant groups. Both groups of patients showed significant improvements in Harris scores (P < 0.05). However, in terms of surgical safety, the dominant group had significantly shorter average operation time and lower blood loss compared to the non-dominant group (P < 0.05). This is because when surgeons have to use their non-dominant hand to perform the operation, it is often more challenging to achieve precise positioning and angles when opening at the proximal end of the femur, and it is also difficult to control the direction and force when inserting the main nail, resulting in the need for repeated actions by the surgeon to achieve the desired outcome. This consumes more surgical time and intraoperative bleeding. Additionally, the dominant group had a higher proportion of satisfactory reduction and fracture stability compared to the non-dominant group, but the difference was not statistically significant (P > 0.05) Just like in our usual surgeries, for elderly IFF patients with osteoporosis, repeated openings and insertions of the main nail can often lead to loss of fracture reduction. Elderly patients with IFF often have a higher mortality rate, so we also focused on the difference in mortality rates between the two groups of patients. According to the 1-year follow-up results, there were 86 survivors and 4 deaths in the dominant group, and 80 survivors and 12 deaths in the non-dominant group. Although the non-dominant group (13.04%) had a higher mortality rate than the dominant group (4.44%), the difference between the two groups was not statistically significant (P > 0.05). In other words, although the handedness of surgeons has a certain impact on the efficacy and safety of the surgery, this impact is not sufficient to affect the mortality rate of patients.

Our findings also have important implications for the training of younger or less experienced surgeons. While the experienced surgeons in our study demonstrated the ability to adapt to the challenges of operating on the non-dominant side, less experienced surgeons may face greater difficulties, potentially leading to longer surgical times, increased blood loss, and less favorable outcomes. To address this, we suggest that surgical training programs incorporate targeted exercises to improve ambidexterity and familiarity with non-dominant side procedures. Simulation-based training, cadaveric workshops, and supervised practice on the non-dominant side could help trainees develop the necessary skills and confidence. Additionally, the use of advanced imaging and navigation technologies during training could provide real-time feedback and enhance precision, regardless of the surgeon’s handedness. Developing proficiency in operating on both sides not only enhances versatility and adaptability in the operating room but may also reduce outcome disparities and contribute to more consistent surgical performance. Future research should evaluate the effectiveness of ambidexterity training programs and their long-term impact on surgical outcomes, particularly in high-risk populations such as elderly patients with intertrochanteric fractures.

This study had some limitations. First of all, this study was a single-center retrospective study, only four surgeons were included, and the sample size of patients was small and the follow-up time was short. More surgeons, more patients, and longer follow-up are needed to add strength to the study. Secondly, this study only compared the stability of fracture reduction and did not study the placement of internal fixation implants. Further improvement in postoperative CT scans is needed to obtain more accurate and comprehensive information. Thirdly, this study did not further exclude other potential confounders that may affect postoperative function, such as the patient’s comorbidities or the severity of osteoporosis. Future studies with more comprehensive data collection, including bone mineral density measurements and detailed comorbidity profiles, would be valuable in further elucidating the impact of these variables.

## Conclusion

The efficacy and safety of PFNA surgery in IFF patients are the result of multiple factors, some related to inherent attributes of the surgeons, and others related to individual characteristics of each case. The results of this study suggest that handedness is one of these factors, particularly concerning the insertion of the main nail. While the observed effects are modest, it has not been previously demonstrated and may be worth considering when performing PFNA surgery with the non-dominant hand.

## Data Availability

The original contributions presented in the study are included in the article/supplementary material, further inquiries can be directed to the corresponding authors.

## Abbreviations

PFNA, proximal femoral nail antirotation; IFF, intertrochanteric fractures of the femur; PMCS, Positive medial cortex support; NP, Neutral position; NMCS, Negative medial cortex support; Hb, hemoglobin.
